# Acute Effects of Different Muscle Contraction Types on Biomechanical and Viscoelastic Properties of the Biceps Brachii Measured with Myotonometry

**DOI:** 10.3390/jfmk11010030

**Published:** 2026-01-08

**Authors:** Sebastian Szajkowski, Jarosław Pasek, Grzegorz Cieślar

**Affiliations:** 1 Faculty of Medical Sciences, Warsaw Medical Academy of Applied Sciences, 8 Rydygiera St., 01-793 Warszawa, Poland; 2 Collegium Medicum, Jan Długosz University in Częstochowa, 13/15 Armii Krajowej St., 42-200 Częstochowa, Poland; 3 Department of Internal Medicine, Angiology and Physical Medicine, Faculty of Medical Sciences in Zabrze, Medical University of Silesia in Katowice, 15 Stefana Batorego St., 41-902 Bytom, Poland

**Keywords:** muscle contraction, muscle fatigue, resistance training, exercise, biomechanical phenomena

## Abstract

**Background:** Acute alterations in biomechanical and viscoelastic muscle properties provide important insight into early fatigue mechanisms; however, their dependence on specific muscle contraction types remains insufficiently understood. Therefore, the aim of this study was to quantitatively compare the acute effects of eccentric, concentric, isometric, and mixed contractions on the biomechanical and viscoelastic properties of the biceps brachii using myotonometry. **Methods:** Eighty healthy men aged 40 to 50 years were randomly assigned to four contraction conditions: eccentric, concentric, isometric or mixed concentric-eccentric. Each participant performed four sets of isolated biceps brachii exercise to volitional failure. Myotonometric measurements of tone, stiffness, decrement, relaxation and creep were collected before exercise and after each set. Changes within and between contraction types were analyzed. **Results:** Muscle responses differed significantly depending on contraction type. Dynamic contractions induced immediate viscoelastic changes, with significant reductions in relaxation time after eccentric (*p* = 0.027), concentric (*p* = 0.026), and mixed contractions (*p* < 0.001), while no changes were observed after isometric contraction (*p* = 0.285). Stiffness remained stable across all contraction types (*p* > 0.05). Mixed contractions showed a biphasic response in decrement with a significant effect across series (*p* = 0.049), identifying decrement as the most sensitive indicator of early fatigue, whereas isometric contraction produced no significant modifications in any parameter. **Conclusions:** Dynamic muscle work induces rapid and contraction-dependent shifts in viscoelastic properties, whereas stiffness appears resistant to short-term loading. Isometric contractions display minimal mechanical disturbance. Myotonometry proved effective in detecting early fatigue-related changes and decrement may serve as a key marker of short-term muscle adaptation.

## 1. Introduction

In recent years, there has been a marked increase in interest among researchers and clinical practitioners in the stiffness of soft tissues, understood as the resistance of biological structures to externally imposed deformation [1]. This parameter has gained prominence due to accumulating evidence indicating its key role in identifying structural abnormalities, assessing the effectiveness of therapeutic or training interventions, and preventing sports-related injuries. Soft tissue stiffness provides valuable insight into the structural and functional properties of muscles and connective tissue. Moreover, various types of muscle contractions have been shown to modulate the level of fatigue, the extent of muscle fiber damage, and post-exercise performance capacity [2,3].

Monitoring exercise induced fatigue is becoming an essential component in the optimization of both training and rehabilitation processes. From the perspective of physiology and sports medicine, it plays an important role not only in enhancing performance but also in preventing overuse injuries. Advances in technology, including methods that assess electrochemical and mechanical properties of muscle such as excitation transmission, propagation of the action potential, and changes in viscoelastic properties within the muscle tendon complex, have significantly improved our understanding of the mechanisms that contribute to muscular fatigue [4].

Among the available tools used to evaluate biomechanical and viscoelastic properties of soft tissues, myotonometry has emerged as an especially valuable method. It enables rapid, noninvasive, and repeatable assessment of muscle tissue characteristics. This technique allows for the detection of subtle biomechanical changes that may serve as early indicators of developing muscle fatigue. This has been confirmed by numerous studies examining responses to a variety of exercise protocols [5,6]. From a clinical point of view, such measurements offer meaningful insights into the mechanisms underlying musculoskeletal dysfunction.

The MyotonPRO, one of the most widely used myotonometers, is a lightweight, portable, and user-friendly device that measures biomechanical and viscoelastic properties of muscles and tendons [7]. Through digital palpation of superficial tissues, the device records mechanically induced oscillations, from which it calculates parameters such as muscle tone, stiffness, decrement, frequency, creep, and relaxation [8,9]. Despite the growing evidence supporting its clinical utility, comprehensive data regarding its measurement properties under conditions of substantial muscle fatigue are still lacking. Such conditions may influence both the repeatability of the measurements and their interpretation [10].

Given these considerations, further investigation of alterations in muscle properties resulting from different activation modalities, as well as the diagnostic relevance of these changes, appears justified. The biceps brachii was selected due to its superficial location, simple fusiform architecture, and frequent use in experimental fatigue studies, which together ensure high measurement reliability, standardized exercise execution, and comparability with existing literature. Therefore, the aim of the present study was to examine and compare the acute effects of isolated resistance exercise of the biceps brachii on mechanical muscle properties assessed by myotonometry and to visualize the variability of fatigue responses across different contraction types. This study seeks to contribute to the expanding knowledge of soft tissue mechanics and its practical applications in medicine, physiotherapy, and sports science.

## 2. Material and Methods

A total of eighty healthy male volunteers (mean age 42.58 ± 2.03 years; mean BMI 28.18 ± 3.31 kg/m^2^) were recruited for the study. Eligibility was restricted to individuals aged 40–50 years with a BMI between 18.5 and 29.9 kg/m^2^, with no contraindications to physical exercise and no history of upper-limb injuries or surgical interventions. All participants reported low habitual physical activity levels and were not involved in structured sports training; they attended a gym no more than one to two times per week. The narrow age and BMI intervals were intentionally selected to limit the confounding effects of age-related morphological or biomechanical alterations in muscle tissue. Participants were excluded if they had sustained any injury requiring treatment within the preceding six months, experienced recent musculoskeletal trauma, reported fatigue or fever, presented with chronic medical conditions, or were taking any medications at the time of enrollment. Furthermore, they were instructed to refrain from any strenuous physical activity for a minimum of 72 h before undergoing the experimental procedures. All volunteers were notified of their right to withdraw from the study at any stage without justification.

### 2.1. Study Design

**Figure 1:**
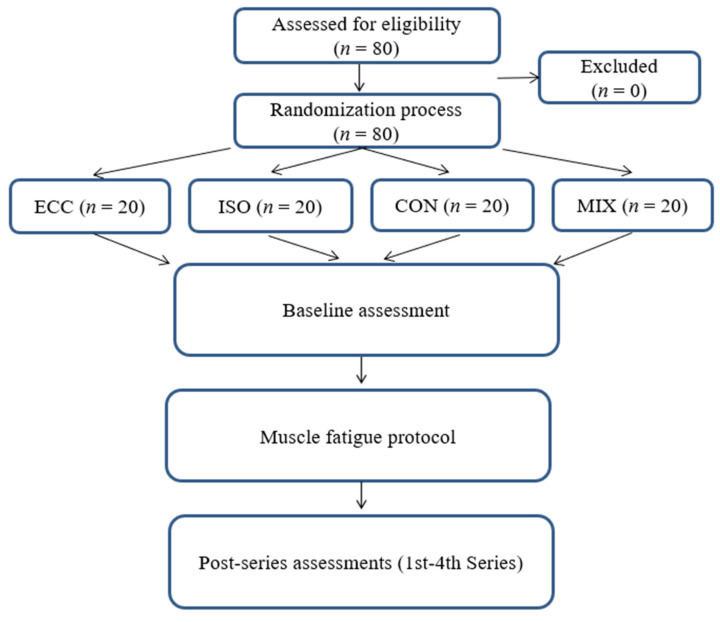
Study design.

Eighty participants were randomly allocated into four experimental groups (*n* = 20 per group): ECC, ISO, CON and MIX, using Microsoft Excel. Randomization was performed by generating a pseudo-random number for each participant with the *RAND()* function, sorting the list in ascending order of these values and then assigning individuals sequentially to the four groups. This procedure ensured an unbiased allocation and eliminated the possibility of predicting group assignment. Muscle fatigue protocol: Each participant completed four sets to volitional muscular failure according to the assigned contraction mode: eccentric only (ECC), isometric (ISO), concentric only (CON) or mixed concentric and eccentric (MIX). Myotonometric measurements were collected at five time points: baseline (pre-exercise) and after each of the four subsequent sets (Figure 1). The research was carried out at the Didactic and Scientific Centre of the Warsaw Medical Academy of Applied Sciences in Warsaw, Poland. All procedures adhered to the ethical principles of the Declaration of Helsinki (1964). The study protocol received approval from the institutional Bioethics Committee (approval ID: 202506WAM04).

### 2.2. Muscle Fatigue Protocol

The intervention consisted of an isolated biceps brachii exercise performed on a preacher (Scott) bench using the dominant upper limb. Each participant completed four consecutive sets to volitional muscular failure, defined as the inability to perform another repetition with correct technique. The assigned contraction mode determined execution: eccentric only (ECC), isometric (ISO), concentric only (CON) or mixed concentric and eccentric (MIX). A supervising physiotherapist monitored all sets and assisted with the load when necessary to ensure strict adherence to the prescribed contraction type and to prevent compensatory movements or momentum. A standardized warm-up was performed prior to the fatigue protocol in accordance with procedures commonly used in resistance-exercise research [5,11]. Participants completed one set of elbow-flexion exercise on the preacher (Scott) bench using a light, submaximal load corresponding to approximately 50 percent of their estimated training load. The warm-up consisted of 10 to 12 controlled repetitions performed at the same tempo that was later used during the experimental sets.

**Figure 2:**
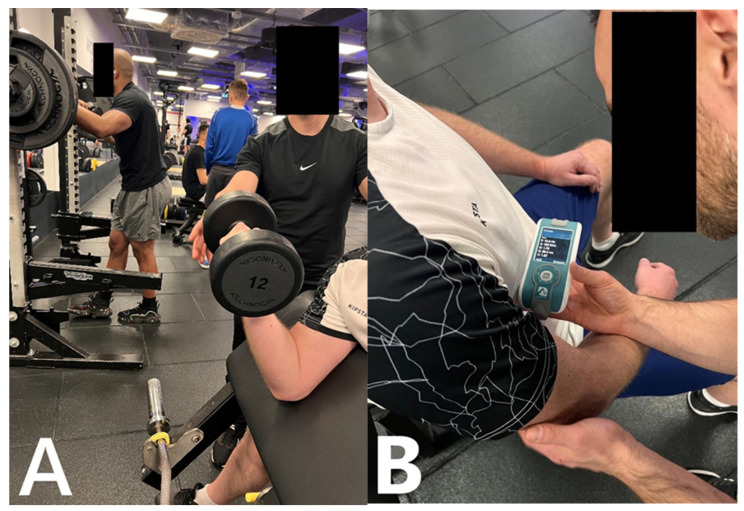
(**A**,**B**). Experimental setup for the biceps brachii fatigue protocol and myotonometric assessment.

Exercise execution was standardized across groups. In the ISO group, an isometric contraction was maintained at approximately 90 degrees of elbow flexion, and each isometric contraction was held for 6 s, which is consistent with commonly used durations of prolonged isometric contractions reported in neuromuscular research [12,13]. In the CON group, the concentric phase was executed actively while the eccentric phase was assisted; in the ECC group, the eccentric phase was active and the return movement assisted. The MIX group performed full-range repetitions including both phases. An individualized submaximal load that allowed repeated sets to failure was selected for each participant, and all performance parameters were documented. The load was predetermined individually so that volitional muscular failure occurred between the tenth and twelfth repetition, ensuring a comparable level of effort across participants (Figure 2A,B).

Exercise tempo was externally controlled using a metronome set to 60 beats per minute (one beat equal to one second). For the CON, ECC and MIX groups, repetitions followed a 3 s concentric phase and a 3 s eccentric phase, yielding a total time under tension of 6 s per repetition. In the ISO group, isometric contractions were held for 3 s [14,15]. Rest intervals between sets were fixed at one minute and timed with a stopwatch. All procedures were conducted under controlled environmental conditions between 10:00 and 14:00, at a constant ambient temperature of 21 °C and a relative humidity of 50 percent.

### 2.3. Myotonometry Assessment

Myotonometric evaluation of the biceps brachii was performed using the MyotonPRO device (Myoton Ltd., Tallinn, Estonia) in accordance with standardized procedures. The measurement point was established at the midpoint between the anterior tip of the lateral acromion and the medial border of the cubital fossa on the dominant upper limb [16] and marked on the skin prior to assessment. All assessments were carried out in a seated position. The examined arm rested on the participant’s thigh with the forearm maintained in a neutral orientation and the elbow positioned in a natural degree of flexion (approximately 90 degrees), (Figure 2A,B) [17].

Measurements were taken before the exercise protocol at baseline and one minute after each completed set. The one-minute interval allowed the muscle tissue to stabilize following exertion, which reduced tremor related and mechanical artifacts while still capturing early exercise induced biomechanical changes [18].

The MyotonPRO is a handheld digital instrument equipped with a 3 mm probe that enables non-invasive analysis of soft-tissue mechanical properties. The device operates on the principle of mechanical impulse response: a brief, controlled tap is applied to the muscle, and the resulting oscillations are captured by an integrated accelerometer. The accelerometer records tissue oscillations at a sampling frequency of 3200 Hz, ensuring high temporal resolution of the mechanical response. From the recorded acceleration and displacement signals, the device calculates key biomechanical parameters, including muscle tone, stiffness, and logarithmic decrement, the latter reflecting elasticity and viscoelastic damping properties. Additional outcomes include mechanical stress relaxation time and the Deborah number, which represents the relation between relaxation time and deformation time and characterizes tissue creep behavior. During each measurement, the probe was positioned perpendicular to the skin at the marked site. The device applied an initial pre-load force of 0.18 N followed by five consecutive mechanical impulses of 0.4 N, each lasting 15 ms, to induce tissue oscillation. All mechanical responses were recorded continuously by the accelerometer [19]. To ensure measurement reliability, the coefficient of variation was monitored for every trial, and any measurement exceeding three percent was repeated. Each parameter was recorded three times, and mean values were used for analysis. A complete measurement sequence did not exceed twenty seconds. All assessments were performed by a physiotherapist experienced in myotonometry and research protocols.

#### Description of Myotonometric Parameters

• Oscillation frequency (F) [Hz] reflects muscle *tone*. Higher values indicate greater intrinsic muscle tension and stiffness.
• Dynamic *stiffness* (S) [N/m] describes the resistance of the tissue to deformation in response to an external force. Higher values indicate a stiffer structure and reduced muscle compliance.
• Logarithmic *decrement* (log) [dimensionless] represents oscillation damping and is inversely proportional to tissue elasticity. Lower values indicate lower damping and greater elasticity, defined as the ability of the tissue to return to its original shape after deformation.
• Mechanical stress *relaxation time* (R) [ms] refers to the time required for the tissue to return to its baseline state following external deformation. Shorter relaxation times indicate greater tissue stiffness and tension, whereas longer relaxation times reflect higher elasticity.
• *Creep* index (C) is a dimensionless parameter, also known as the Deborah number, defined as the ratio of relaxation time to deformation time. It characterizes creep behavior, which refers to the gradual elongation of tissue under constant load. Lower values indicate more elastic tissue properties, while higher values reflect increased stiffness [8,9,10,18,19].

### 2.4. Statistics

Data distribution was assessed using the Shapiro–Wilk test, and the homogeneity of variance across groups was verified with Levene’s test. Descriptive statistics are presented as means and standard deviations. To examine changes in biomechanical and viscoelastic muscle properties across the four consecutive loading series within each contraction type (ECC, ISO, CON, MIX), a one-way repeated measures analysis of variance (ANOVA) was conducted separately for each dependent variable (tone, stiffness, decrement, relaxation, and creep). The repeated measures ANOVA was used as a global test of the series effect within each contraction modality. When the assumption of sphericity was violated, the Greenhouse–Geisser correction was applied. To analyze between-group differences among the four contraction types at each measurement time point (baseline and after each series), a one-way ANOVA was performed independently for each variable. In both within-group and between-group analyses, post hoc comparisons were conducted only when a significant main effect was detected, using Bonferroni-adjusted tests to control for inflation of Type I error due to multiple comparisons. Thus, multiple pairwise comparisons were not treated as independent tests with manually adjusted significance thresholds; instead, error control was ensured through the use of global ANOVA models followed by Bonferroni-corrected post hoc procedures. Statistical significance was defined as *p* < 0.05 for the global ANOVA tests. All statistical analyses were performed using PQStat software (version 1.8.6).

Sample size estimation was based on the decrement parameter, which demonstrated the greatest between-group variability in preliminary observations. Using the estimated effect size derived from mean and standard deviation values in the first loading series (Cohen’s f = 0.32), a four-group one-way ANOVA with α = 0.05 and statistical power of 0.80 indicated a minimum required sample size of 76 participants. To account for potential dropouts, the target sample size was increased by approximately 10%, resulting in a final sample size of 80 participants, which matched the number of subjects included in the study. Sample size calculations were performed using G*Power software (version 3.1.9.7; Heinrich-Heine-Universität Düsseldorf, Düsseldorf, Germany) [20].

## 3. Results

**Figure 3:**
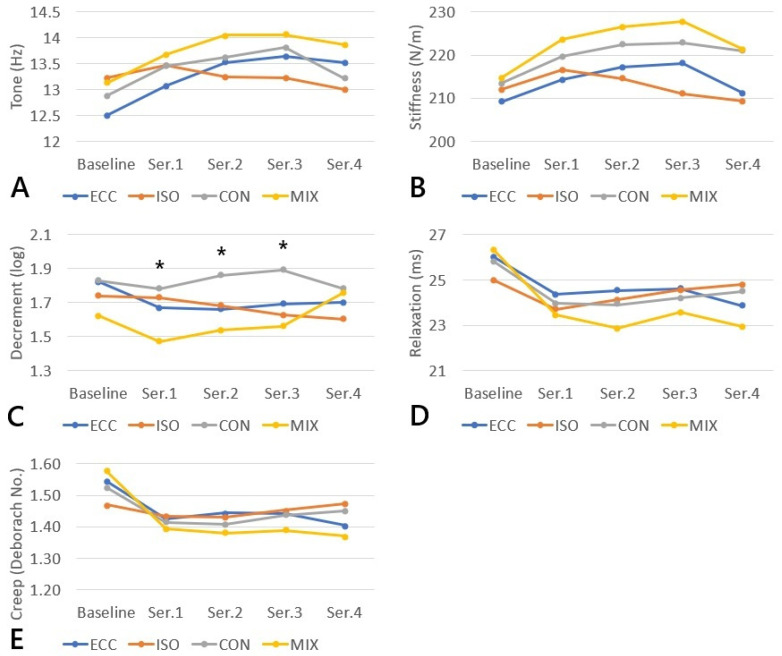
(**A**–**E**). Changes in biomechanical and viscoelastic muscle parameters across consecutive loading series for different types of muscle contraction. ((**A**)—Tone; (**B**)—Stiffness; (**C**)—Decrement; (**D**)—Relaxation; (**E**)—Creep). Statistically significant differences (*p* < 0.05) between groups are indicated with *.

The analysis of biomechanical and viscoelastic muscle parameters in response to consecutive loading series revealed distinct patterns of change depending on the type of muscle contraction. The results are presented in Table 1A–E and Figure 3A–E. For *tone*, a significant series effect was observed only in the eccentric contraction (*p* = 0.014), whereas isometric, concentric, and mixed contractions did not demonstrate significant changes across series (*p* = 0.547, 0.157, and 0.124, respectively). No significant between-group differences were identified in any series.

For *stiffness*, no significant changes across series were detected for any contraction type (*p* = 0.069–0.317), nor were significant between-group differences observed at any measurement point.

The *decrement* parameter, which inversely reflects muscle elasticity (with lower values indicating greater elasticity), exhibited significant changes in the mixed contraction (*p* = 0.049), characterized by a reduction in the initial series followed by an increase in the fourth series. Additionally, significant between-group differences were noted in series 1–3: series 1: *p* = 0.03 (ISO vs. MIX; CON vs. MIX), series 2: *p* = 0.021 (ECC vs. CON; ISO vs. CON; CON vs. MIX), series 3: *p* = 0.042 (ECC vs. MIX; CON vs. MIX). No significant series effects were observed in the remaining contraction modalities.

For *relaxation*, a significant reduction in relaxation time was identified in the eccentric (*p* = 0.027), concentric (*p* = 0.026), and mixed contractions (*p* < 0.001). The isometric contraction did not show significant changes (*p* = 0.285). No significant between-group differences were found in any series.

The *creep* parameter demonstrated significant series effects in the eccentric (*p* = 0.021) and concentric contractions (*p* = 0.032), while in the mixed contraction the trend did not reach statistical significance (*p* = 0.359). Values remained stable throughout the isometric contraction. No significant between-group differences were observed for *creep* at any measurement point.

## 4. Discussion

The results of the present study demonstrate that the muscle response to loading is clearly dependent on the type of contraction, with isometric contraction exhibiting the highest biomechanical and viscoelastic stability compared with dynamic contractions. These findings are consistent with numerous previous reports indicating that dynamic forms of muscle work, particularly eccentric and mixed (concentric-eccentric) contractions, disrupt mechanical homeostasis to a much greater extent than static work. In the mixed contraction condition, decrement showed significant changes both between sets and between the individual contraction types. In our study, immediate changes in relaxation, creep, and decrement were observed exclusively following dynamic contractions, confirming that viscoelastic properties are sensitive to short-term mechanical stimuli and may serve as sensitive markers of early muscle fatigue.

The results obtained in the present study following eccentric loading of the muscle align with the findings reported by Kawczyński et al. [21] who demonstrated that eccentric contractions lead to heterogeneous and often delayed alterations in muscle stiffness and creep. Although their study primarily reported changes occurring 24 h post-exercise, our results document a much earlier, immediate phase of adaptation, emerging already from series to series. Further support for this interpretation is provided by the findings of Lall et al. [22], who showed that eccentric loading induces a rapid increase in Achilles tendon stiffness and in the medial head of the gastrocnemius muscle, with these changes varying across different muscle regions. Our interpretation is further corroborated by the work of Klich et al. [23] who reported that intense sprint and endurance exercise induces rapid, segment-specific alterations in quadriceps stiffness, with movement specificity (corresponding to contraction type) serving as a key determinant of regional tissue responses. Similar observations were presented in the study by Chang et al. [24], where rowing exercise resulted in increased stiffness of the shoulder-girdle musculature and the rectus femoris.

An important observation in our study is the confirmation that parameters such as relaxation, creep, and decrement are considerably more reactive to loading than stiffness. According to the findings of Krzysztofik et al. [25], oscillation frequency (and thus properties dependent on passive tension) increases immediately after supramaximal exertion, whereas stiffness remains unchanged, which was interpreted as a marker of early fatigue. Similar phenomena were observed in our data, where stiffness remained stable across all contraction types, while viscoelastic parameters (relaxation and creep) exhibited dynamic alterations. The results of Lettner et al. [26] further support this mechanism, showing that fatigue of the vastus lateralis and medialis muscles resulted in delayed increases in stiffness, whereas changes in viscoelastic parameters appeared immediately. The findings regarding decrement, which in our study emerged as one of the most sensitive indicators of adaptation, are fully consistent with reports on delayed-onset muscle soreness (DOMS) and recovery interventions. In the study by Szajkowski et al. [27], which analyzed acute changes in biomechanical and viscoelastic muscle properties following concentric and eccentric loading performed to volitional muscular failure, several myotonometric parameters were found to respond to exercise induced fatigue. Decrement emerged as a sensitive marker of load related alterations, whereas stiffness remained relatively stable and did not demonstrate immediate fatigue associated changes. A comparable pattern was observed in studies investigating recovery interventions applied after exercise inducing delayed onset muscle soreness [28]. Following a standardized fatigue protocol performed to muscular failure, changes in myotonometric parameters were noted during the recovery period, with decrement showing significant modulation, particularly in response to foam rolling, while stiffness exhibited more limited or delayed alterations. Similarly, in a study examining the effects of thermal interventions such as infrared radiation and local cryotherapy applied after exercise to muscular failure [29], changes in elasticity related parameters including decrement were observed during recovery, whereas alterations in stiffness were less pronounced and occurred later in the observation period. These observations confirm that decrement may be one of the most useful parameters for monitoring early changes in muscle elasticity. It is worth emphasizing that a similar pattern of viscoelastic response has been reported in cycling and high-intensity exercise. Rotllan et al. [30] demonstrated that following a cycling race, muscle stiffness and tone decreased while elasticity increased, which was interpreted as a transient viscoelastic reorganization resulting from accumulated fatigue. In our study, mixed contractions exhibited a similar biphasic response: a decrease in decrement (i.e., an increase in elasticity) in the initial series, followed by an increase in this parameter in the later part of the protocol.

Taken together, the present findings, along with those reported in the literature, clearly indicate that immediate viscoelastic changes in muscles are specific to and dependent on the type of muscle contraction, with a pronounced dominance of responses during dynamic contractions. The stability of isometric parameters aligns with all existing evidence, emphasizing that isometric work minimally perturbs tissue mechanics. The active muscle response is spatially heterogeneous and depends on both local tissue properties and the nature of force generation. Early alterations in relaxation, creep, and decrement may serve as highly sensitive markers of fatigue and muscle fiber reorganization, whereas stiffness remains a more stable structural parameter, responding primarily over a longer time scale. Thus, the integration of these findings demonstrates that the muscle response to loading is rapid, complex, and contraction-specific, and that short-term adaptation is best reflected by viscoelastic properties.

## 5. Conclusions

The study confirmed that the acute response of the biceps brachii to loading depends primarily on the type of contraction. Dynamic contractions, including eccentric, concentric, and mixed forms, produced immediate and clearly detectable changes in viscoelastic properties, particularly in relaxation, creep, and decrement. These findings indicate that these parameters are highly sensitive to early stages of fatigue. The mixed contraction revealed the most complex response pattern, showing significant changes across subsequent series as well as differences compared with other contraction types, which highlights the complexity of adaptive mechanisms occurring during alternating phases of movement. In contrast, the isometric contraction displayed biomechanical stability and did not show significant changes in any of the analyzed parameters. This confirms its distinct characteristics and its limited influence on short-term reorganization of muscle tissue. The stiffness parameter proved to be the least responsive to brief loading, suggesting that rapid fatigue-related changes occur mainly within viscoelastic rather than structural properties.

Overall, the results indicate that monitoring the complete biomechanical profile of the muscle, especially decrement as an inverse indicator of elasticity, may serve as a valuable tool for assessing fatigue, adaptation, and readiness for loading. The findings also confirm the usefulness of myotonometry for evaluating short-term muscular adaptations and emphasize the dominant role of contraction type in shaping the immediate mechanical response of muscle tissue.

### Limitations of the Study

This study has several limitations that restrict the generalizability of the findings to a broader population. First, the sample was demographically specific and limited to middle-aged men (40–50 years old). While the applied inclusion criteria allowed the formation of a homogeneous group, increasing the reliability and internal consistency of the results, they also substantially limited external applicability. Consequently, the findings may not be directly generalizable to women, elite athletes, or older adults, in whom differences in hormonal profiles, training status, and muscle architecture may influence mechanical and fatigue-related responses.

Biomechanical and viscoelastic properties were measured at a single predetermined point on the muscle, which may not capture regional variations within the biceps brachii or fully represent its overall functional characteristics. In addition, the biceps brachii is a superficial, fusiform muscle, and the results observed in this muscle may not be transferable to deeper or pennate muscles (e.g., the gastrocnemius), which exhibit different architectural and mechanical properties. Another limitation is the lack of concurrent assessment modalities. Although myotonometry is a valid and effective method for evaluating muscle mechanical properties, the absence of electromyographic (EMG) measurements or metabolic markers (such as blood lactate concentration) precludes a comprehensive correlation between observed mechanical changes and underlying neuromuscular activation or biochemical mechanisms of fatigue.

The exercise protocol was performed on a preacher bench, which is more representative of real training conditions but more difficult to standardize compared to isokinetic testing. Although isokinetic dynamometry allows for precise control of contraction velocity and movement phases, such movements are unfamiliar and non-naturalistic for most individuals. For the purposes of this study, stable limb positioning and a strictly repeatable exercise protocol were considered sufficient to ensure methodological consistency.

Future research should include more diverse participant groups, assess muscles with different architectural characteristics, and incorporate complementary physiological measures. It would also be valuable to evaluate muscle responses both immediately after exercise and during later stages of recovery in order to obtain a more comprehensive understanding of muscle response dynamics.

## Figures and Tables

**Table 1:** (**A**–**E**). Biomechanical and viscoelastic muscle parameters across consecutive loading series for different types of muscle contraction. ((**A**)—Tone; (**B**)—Stiffness; (**C**)—Decrement; (**D**)—Relaxation; (**E**)—Creep).

(A)	Baseline	Ser. 1	Ser. 2	Ser. 3	Ser. 4	
**Tone (Hz)**	mean ± SD	mean ± SD	mean ±SD	mean ± SD	mean ± SD	*p* (between series)
ECC	12.5 ± 1.4	13.07 ± 1.84	13.53 ± 1.69	13.64 ± 2.01	13.52 ± 1.48	**0.014**
ISO	13.23 ± 1.86	13.47 ± 1.26	13.24 ± 1.57	13.23 ± 1.1	13 ± 1.06	0.547
CON	12.88 ± 2.01	13.45 ± 1.46	13.62 ± 1.35	13.81 ± 1.63	13.21 ± 1.48	0.157
MIX	13.13 ± 1.64	13.67 ± 1.18	14.05 ± 1.55	14.06 ± 1.52	13.86 ± 1.36	0.124
*p* (between groups)	0.558	0.622	0.431	0.218	0.443	
**(B)**	**Baseline**	**Ser. 1**	**Ser. 2**	**Ser. 3**	**Ser. 4**	
**Stiffness (N/m)**	mean ± SD	mean ± SD	mean ± SD	mean ± SD	mean ± SD	*p* (between series)
ECC	209.25 ± 23.45	214.4 ± 30.84	217.25 ± 32.21	218 ± 33.98	211.25 ± 30.77	0.22
ISO	212 ± 22.94	216.65 ± 28.49	214.6 ± 30.62	211.1 ± 21.01	209.3 ± 16.05	0.317
CON	213.5 ± 26.71	219.75 ± 26.9	222.45 ± 24.56	222.9 ± 21.31	221.05 ± 22.03	0.245
MIX	214.75 ± 16.88	223.65 ± 20.7	226.45 ± 28.37	227.75 ± 28.56	221.3 ± 25.59	0.069
*p* (between groups)	0.885	0.724	0.775	0.285	0.168	
**(C)**	**Baseline**	**Ser. 1**	**Ser. 2**	**Ser. 3**	**Ser. 4**	
**Decrement (log)**	mean ± SD	mean ± SD	mean ± SD	mean ± SD	mean ± SD	*p* (between series)
ECC	1.82 ± 0.3	1.67 ± 0.2	1.66 ± 0.31	1.69 ± 0.22	1.7 ± 0.25	0.165
ISO	1.74 ± 0.34	1.73 ± 0.3	1.68 ± 0.23	1.63 ± 0.29	1.6 ± 0.28	0.168
CON	1.83 ± 0.23	1.78 ± 0.24	1.86 ± 0.3	1.89 ± 0.31	1.78 ± 0.29	0.311
MIX	1.62 ± 0.3	1.47 ± 0.23	1.54 ± 0.26	1.56 ± 0.24	1.76 ± 0.36	**0.049**
*p* (between groups)	0.748	**0.03**	**0.021**	**0.042**	0.501	
**(D)**	**Baseline**	**Ser. 1**	**Ser. 2**	**Ser. 3**	**Ser. 4**	
**Relaxation (ms)**	mean ± SD	mean ± SD	mean ± SD	mean ± SD	mean ± SD	*p* (between series)
ECC	26.02 ± 2.31	24.36 ± 3.24	24.52 ± 3.51	24.59 ± 2.86	23.86 ± 3.19	**0.027**
ISO	24.98 ± 2.8	23.69 ± 2.8	24.11 ± 3.34	24.55 ± 1.97	24.8 ± 1.82	0.285
CON	25.81 ± 3.48	23.96 ± 2.74	23.91 ± 2.44	24.19 ± 2.65	24.47 ± 2.64	**0.026**
MIX	26.33 ± 2.97	23.44 ± 2.33	22.86 ± 2.56	23.58 ± 2.85	22.93 ± 2.96	**<0.001**
*p* (between groups)	0.510	0.754	0.346	0.588	0.145	
**(E)**	**Baseline**	**Ser. 1**	**Ser. 2**	**Ser. 3**	**Ser. 4**	
**Creep (Deborach No.)**	mean ± SD	mean ± SD	mean ± SD	mean ± SD	mean ± SD	*p* (between series)
ECC	1.55 ± 0.13	1.43 ± 0.19	1.44 ± 0.02	1.44 ± 0.15	1.41 ± 0.17	**0.021**
ISO	1.47 ± 0.17	1.43 ± 0.16	1.43 ± 0.21	1.45 ± 0.14	1.47 ± 0.12	0.474
CON	1.53 ± 0.21	1.41 ± 0.15	1.41 ± 0.14	1.44 ± 0.16	1.45 ± 0.16	**0.032**
MIX	1.58 ± 0.18	1.39 ± 0.14	1.38 ± 0.14	1.39 ± 0.16	1.37 ± 0.017	0.359
*p* (between groups)	0.291	0.864	0.382	0.553	0.539	

## Data Availability

The raw data supporting the conclusions of this article will be made available by the authors on request.
